# Narrowing yield gaps does not guarantee a living income from smallholder farming–an empirical study from western Kenya

**DOI:** 10.1371/journal.pone.0283499

**Published:** 2023-04-20

**Authors:** Wytze Marinus, Katrien Descheemaeker, Gerrie W. J. van de Ven, Bernard Vanlauwe, Ken E. Giller

**Affiliations:** 1 Plant Production Systems Group, Wageningen University & Research, Wageningen, The Netherlands; 2 IITA, Central Africa Hub Office, Nairobi, Kenya; Soil and Water Resources Institute ELGO-DIMITRA, GREECE

## Abstract

Crop yields in sub-Saharan Africa need to increase to keep pace with food demands from the burgeoning population. Smallholder farmers play an important role in national food self-sufficiency, yet many live in poverty. Investing in inputs to increase yields is therefore often not viable for them. To investigate how to unlock this paradox, whole-farm experiments can reveal which incentives could increase farm production while also increasing household income. In this study we investigated the impact of providing farmers with a US$ 100 input voucher each season, for five seasons in a row, on maize yields and overall farm-level production in two contrasting locations in terms of population density, Vihiga and Busia, in western Kenya. We compared the value of farmers’ produce with the poverty line and the living income threshold. Crop yields were mainly limited by cash constraints and not by technological constraints as maize yield immediately increased from 16% to 40–50% of the water-limited yield with the provision of the voucher. In Vihiga, at best, one-third of the participating households reached the poverty line. In Busia half of the households reached the poverty line and one-third obtained a living income. This difference between locations was caused by larger farm areas in Busia. Although one third of the households increased the area farmed, mostly by renting land, this was not enough for them to obtain a living income. Our results provide empirical evidence of how a current smallholder farming system could improve its productivity and value of produce upon the introduction of an input voucher. We conclude that increasing yields of the currently most common crops cannot provide a living income for all households and additional institutional changes, such as alternative employment, are required to provide smallholder farmers a way out of poverty.

## Introduction

Crop yields must increase in sub-Saharan Africa (SSA) to keep pace with the food demands of the growing population, to preserve important natural ecosystems and to achieve food self-sufficiency at national and regional level [1–3]. Yields of major cereals have increased only moderately over the past decades, reaching generally only about 20% of the water-limited yield. If current trends continue, SSA cannot achieve self-sufficiency in food by 2050, which would require narrowing the yield gap to at least 50% of the water-limited yield [2]. Smallholder farmers currently contribute about 70% of the national food production [4]. For the required yield increases however, their farming objectives may not match national production goals [5], as food and income generation to meet family needs prevail. Small farm areas represent an important limitation for farmers to realise significant additional farm revenue through investing in farming [6, 7]. Moreover, limited and risky returns on investment act as a disincentive to purchase inputs such as fertilisers [8, 9].

The farmers’ perspective is often overlooked in studies that analysed interventions designed to increase production, e.g. in Ethiopia [10, 11]. At the same time, empirical farm-level studies that try to identify options for improvement often operate within the boundaries imposed by current constraints [12, 13], which limits the ‘solution space’ [14]. On-farm experiments at field level, often in researcher-managed plots, have shown repeatedly that by increasing input use strong increases in crop yields are technically feasible [15–17]. Smallholder farms often show large differences in soil fertility, in crop productivity [18, 19], and in yield responses to inputs, such as fertiliser [20–22]. Part of these differences are explained by the large diversity between households within farming communities in terms of income, farm area and other characteristics [23]. However, there is a scarcity of information on farmers’ decisions on input use and the effects on yield over multiple seasons [24]. Moreover, few empirical examples show whether it is possible to increase yields in all fields of a farm [25] and what would be needed to stimulate increased input use. Input subsidies, e.g. through vouchers, are one option to alleviate household financial constraints for buying inputs and have become common in the past two decades across SSA [26]. They mostly focus on inputs for maize or other important staple crops and aim to reduce poverty and raise household income through increasing production [26] .

The overall aim of our study was to observe and understand diverse farmers’ responses over multiple seasons to provision of input vouchers. Each farmer received a voucher worth US$100 which they could spend on agricultural inputs supplied by the project. We monitored farmer responses and the impacts of the vouchers on farm productivity and income. Rather than comparing their income with the poverty line, which covers the bare minimum needed to live, we used another benchmark of a living income. The living income benchmark considers the income needed for a ‘decent living’ [27, 28]. Our specific objectives were: 1) To assess the impact on maize yield and overall farm-level production of providing a US$100 input voucher during five seasons; 2) To assess whether the changes in production are sufficient to lift a household out of poverty or provide a living income; 3) To assess the extent to which land available for cropping constrains overall production.

## Materials and methods

### Study area

The study took place in two locations in western Kenya. Vihiga county is a typical highland area, with a population among the densest in SSA, ∼1050 people km^-2^, and farm areas covering less than 0.5 ha. Busia county is a medium altitude area with a moderate population density, ∼550 people km^-2^, and farm areas around 1.0 ha [29, 30]. Both locations have a bi-modal rainfall pattern typical of the East-African Highlands, with two cropping seasons per year. The long rains (LR) last from March until June and the short rains (SR) from September until November. Total rainfall in both locations is 1800–2000 mm year^-1^ [31, 32]. Maize is the most important staple crop. It is often intercropped with common bean and both crops together cover about 50% of the farm area. A more detailed description of the study area is given by Marinus et al. [33].

### The input voucher

An input voucher was issued in five subsequent seasons, from 2016SR season until 2018SR. A workshop was organised before each season in which farmers could select agricultural inputs from a list to a maximum value of US$ 100. The value was based on the maximum first loan farmers could obtain from One Acre Fund (OAF). After repayment of the first loan, the maximum amount increased to US$ 270 per season [34]. OAF is a social enterprise providing inputs on credit to farmers in the region (www.oneacrefund.org). Based on farmers’ feedback and researchers’ observations, different inputs were added to the list over time [33]. The inputs included maize, groundnut, soybean, common bean and sorghum seed, mineral fertiliser (diammonium phosphate (DAP), calcium ammonium nitrate (CAN) and Sympal legume fertiliser, soybean inoculant (Biofix, MEA Ltd—Kenya), and other inputs [33]. About half of these inputs were commonly available at agro-input dealers in towns frequented by the farmers. The other inputs were sourced from other places in western Kenya. All inputs were delivered to the farmers by the project. Farmers used on average 80–95% of the voucher value on inputs for maize, groundnut, soybean and common bean [33].

In both Vihiga and Busia two sub-locations with 11–12 farmers each were purposely selected to represent the diversity of farmers in the area from an earlier random survey [33]. Farmers in one of the two sub-locations took part in a co-learning trajectory. All farmers received the same voucher and there were no significant differences in grain yields and income from farming between the sub-locations [33]. Participating farmers were well informed about the purpose of the study prior to participation and informed consent was obtained from all participants involved in the study. Participants were free to withdraw from the study at any moment. Data was collected and stored according to the data management plan of the Plant Production Systems group of Wageningen University (https://git.wur.nl/pps/PPS_data_management/-/raw/master/writing/PPS_Data_Management.pdf). Ethical approval for this study was not required according to the checklist of the Social Sciences Ethics Committee of Wageningen University.

### Detailed farm characterization and farm monitoring

Detailed data on farm productivity and farm management were collected for seven seasons. During the two seasons prior to issuing the input voucher (2015SR and 2016LR) data were collected using a detailed farm characterization survey (DFC, S1 Appendix), following the approach described by Giller et al. [23]. During the first survey, general questions were asked, such as household size and composition, and a map of the farm was drawn. During a second visit, all fields were visited and data on field management and production were collected. Field size was measured using a hand-held GPS or using a tape measure in case of fields with sides less than 20 m.

The same researcher visited all fields during each of the five seasons when farmers received the voucher. During a mid-season visit he observed the crops cultivated and asked about input use in each field. Grain yields of the voucher crops–maize, groundnut, soybean and common bean–were assessed by means of crop cuts. Two 4 × 4 m (16 m^2^) quadrats were placed in each field. Fresh cob (maize) and pod (legumes) yields were measured in the field, and one sub-sample per quadrant was taken to determine oven dry weight. Dry weights were calculated back to a standardized moisture content of 14% and the grain yield (kg ha^-1^, referred to as ‘yield’ hereafter) per field was calculated as the average of the two quadrats. The farm-level yield (kg ha^-1^) per crop was calculated as a weighted average of the fields containing that crop relative to the total area of that crop per farm.

Farm area was monitored throughout the intervention. If fields were added to a farm, it was noted whether these new fields were bought, borrowed, rented-in or whether this was family land that was now used by the household while earlier being lent or hired out. Although detailed data was collected over seven seasons, the limited number of farmers per location precluded a formal statistical analysis.

### Indicators and benchmarks

#### Water-limited yield

Maize yields were compared to the average water-limited yield from the Global Yield Gap Atlas (GYGA) for the Kakamega climate zone, covering both Vihiga and Busia. Those water-limited yields were converted to a moisture content of 14%. For the short rainy season the water-limited yield was 8.0 t ha^-1^ and for the long rainy season, 12.5 t ha^-1^ [35].

#### Value of produce, poverty line and living income

The farm-level value of produce (named value of produce hereafter) was calculated as the measured crop production per season of all fields containing maize, groundnut, soybean and common bean multiplied by their respective median prices for 2018. The median price was assessed through a weekly market survey in both sites. We used the median crop price of 2018 across both sites as prices hardly differed during the season (S2 Appendix). The value of produce was expressed per adult equivalent per day based on the household composition in 2018, following OECD [36] and Van de Ven et al. [28], and the proportional contribution of the short and the long rains cropping seasons to the annual production. Input costs were not considered as these were largely covered by the voucher. The value of produce calculated therefore paints a relatively optimistic figure and does not necessarily reflect profitability of the farm. The poverty line was based on The World Bank [37] and the living income on Anker and Anker [38]. Both were corrected for inflation, using 2018 as reference year, similar as for the crop prices. Both the poverty line and the living income were expressed in Kenya Shilling per adult equivalent.

## Results

### Farm-level maize yields and input use

**Fig 1:**
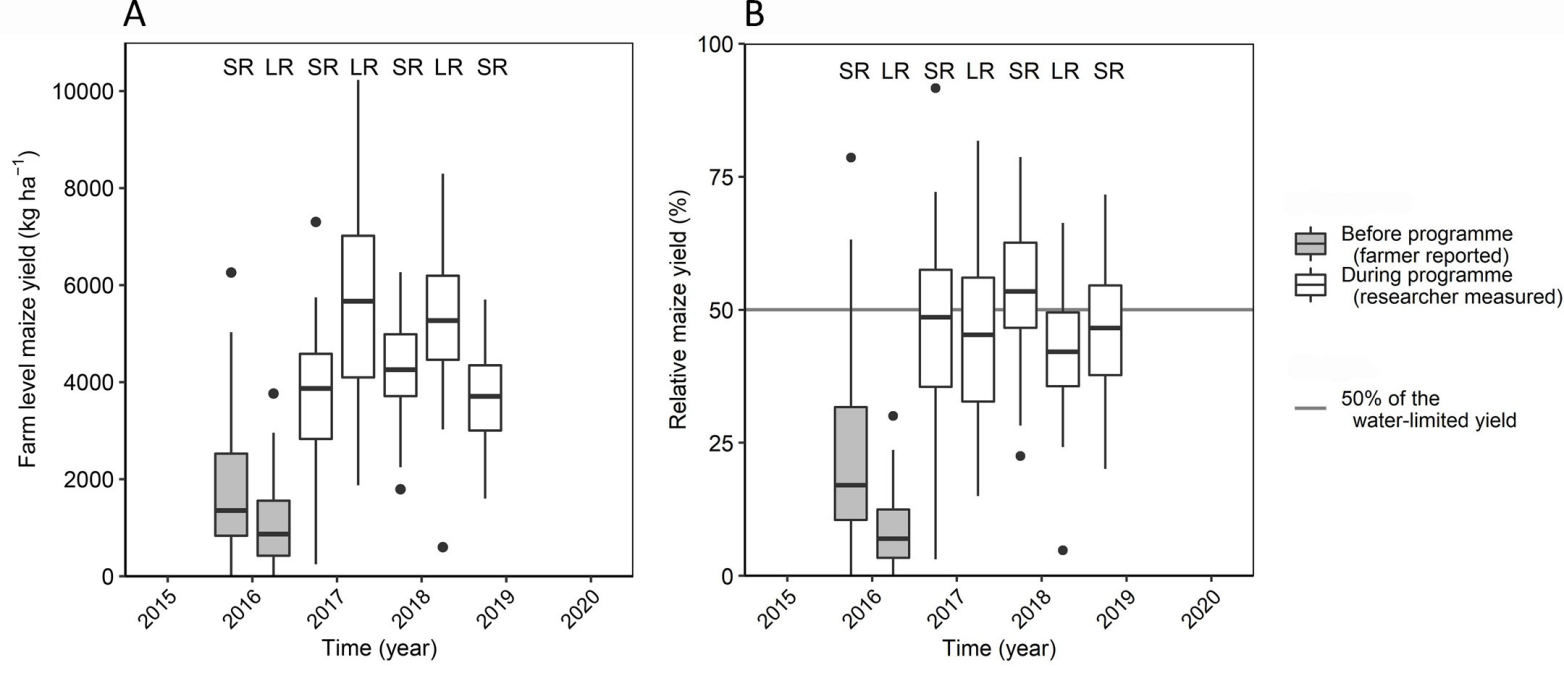
Farm-level maize yields in absolute values (A) and relative to the water-limited yield for the short (SR) and long rain (LR) cropping seasons (B) (n = 47 households with 1–8 maize fields per household). Yields before the programme were based on farmer-reported production and measured field sizes, while during the programme with the voucher available both were measured by researchers. The horizontal line indicates 50% of the water-limited yield.

The maize yield averaged across the farms, increased from 1350 kg ha ^-1^ (2015SR) and 850 kg ha^-1^ (2016LR) before the voucher was introduced, to 3800 kg ha^-1^ and 5400 kg ha^-1^ for the short and the long rains respectively, after introduction of the voucher (Fig 1A). Yields before voucher introduction showed a wide variation, with very low yields in the 2016LR season due to drought. We therefore used 2015SR as the reference season for yields before the programme. The median yields obtained in 2015SR were equivalent to 16% of the water-limited yield. Hence, maize yields increased from less than 16% of the water-limited yield before the interventions to 40–50% of the water-limited yield during the programme (Fig 1B). This increase occurred immediately in the first season the voucher was issued, with no further increase in subsequent seasons.

Farmers improved crop management and increased input use with the introduction of the voucher (Table 1). Nearly all of the maize area per farm was planted with hybrid or improved maize varieties during the programme, while before the programme 53% and 34% of the maize area was planted with local open-pollinated varieties in Vihiga and Busia respectively. Average application rates of mineral fertiliser per season on maize increased for P in Vihiga from 29 to 47 kg P ha^-1^. In Busia N application rates increased from 38 kg N ha^-1^ before to 54 kg N ha^-1^ during the programme and P application rates increased from 19 kg P ha^-1^ before to 25 kg P ha^-1^ during the programme. Moreover, total N and P application per season at farm-level doubled in both locations. However, as maize area per farm also more than doubled in both locations, the increase in total N and P application did not nessesarily lead to a doubling of application rates. For instance, during the programme the N application rates in Vihiga were comparable to the rates applied before the programme.

**Table 1:** Average farm-level maize input use and productivity in the two seasons before the programme and the five seasons during the programme.

		Vihiga		Busia	
		Before programme^1^	During programme	Before programme^1^	During programme
Maize area (ha)		0.13	0.23	0.35	0.51
Maize variety type	Hybrid	44	94	49	92
(% cultivated area)	Improved^2^	2	0	14	0
	Improved OPV^3^	0	2	0	4
	Local OPV	53	3	34	4
Mineral fertiliser	N	94	99	38	54
application rates (kg ha^-1^)	P	29	47	19	25
Mineral fertiliser use	N	9	17	12	24
(kg farm^-1^)	P	3	8	6	11

^1^ Maize varieties, mineral fertilizer use and maize production before the programme were farmer reported, while field sizes were measured during the detailed farm characterisation to calculate farm area, maize area, mineral fertilizer application rates and maize yields. During the programme researchers monitored input use per field and measured maize yields.

^2^ The maize variety category “Improved” was only used during the initial detailed farm characterisation and could include both hybrid and improve open pollinating varieties (OPVs).

^3^ OPV: open pollinating variety

### Value of produce

**Fig 2:**
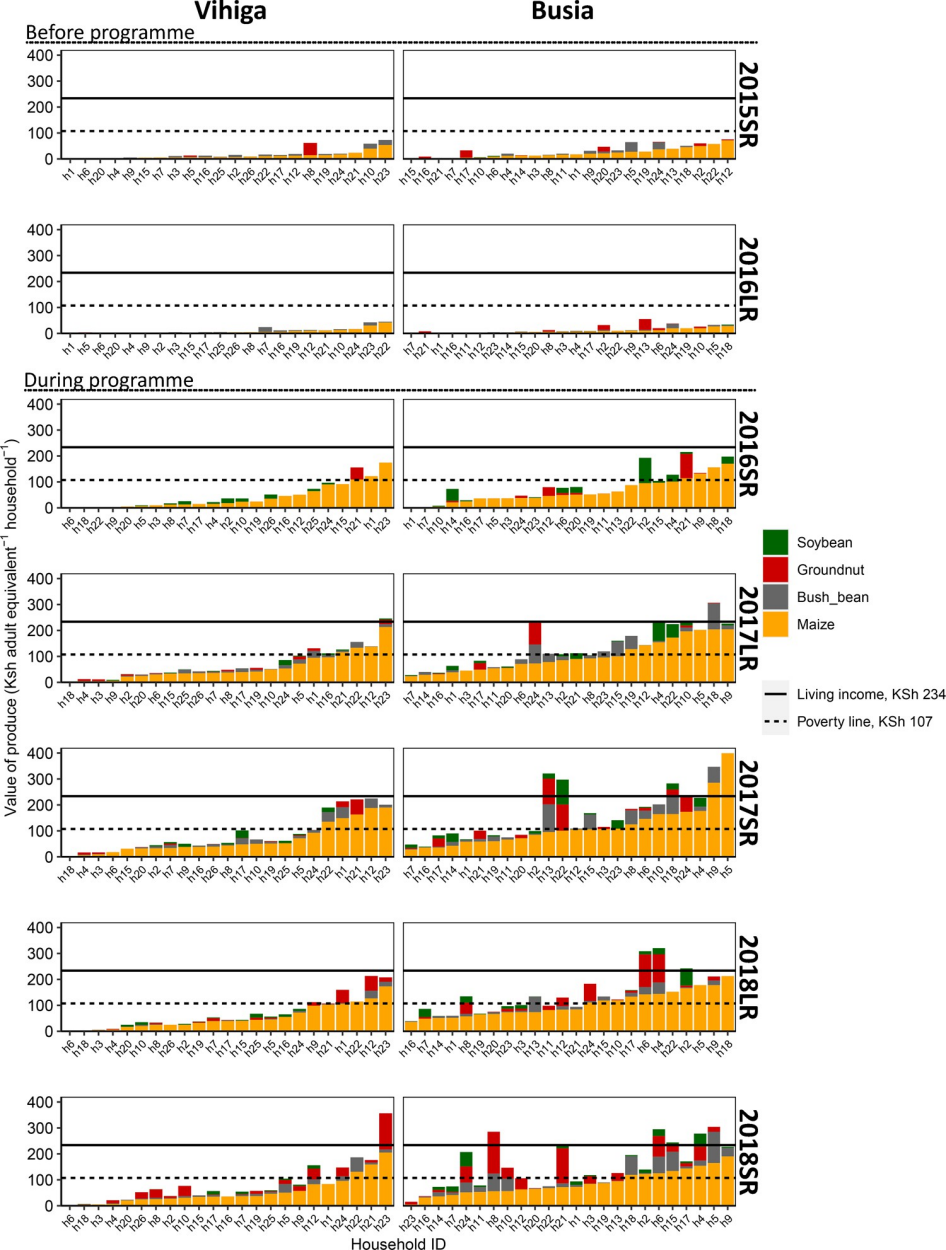
Value of produce in Kenyan Shilling (KSh) per adult equivalent per day for each household. Households were ordered each season based on their value of produce of maize. Household ID’s were assigned per location. Seasons 2015SR and 2016LR were before the programme (farmer-reported production), seasons 2016SR, 2017LR, 2017SR, 2018LR and 2018SR were during the programme with the voucher available (researcher measured production). SR: short rains cropping season; LR: long rains cropping season.

Value of produce per adult equivalent more than tripled from the first season with the voucher onwards, compared to the seasons without a voucher (Fig 2). This was mainly a result of the threefold increase in yield. An increase in cropland allocation to the four voucher crops from about 40–50% to 60–70% of the farm area resulted in an additional increase from the second season onwards. The value of produce in two-thirds of the households in Vihiga never reached the poverty line and it was above the living income threshold for only one of the households in some seasons. In Busia the value of produce from the four crops was higher than the poverty line for half of the households and above the living income threshold for about one quarter of the households in two out of five seasons. Groundnut, common bean and, to a lesser extent, soybean were important crops in terms of value of produce, in particular in Busia. The value of produce of maize alone was sufficient for two households in Busia to achieve a living income.

**Fig 3:**
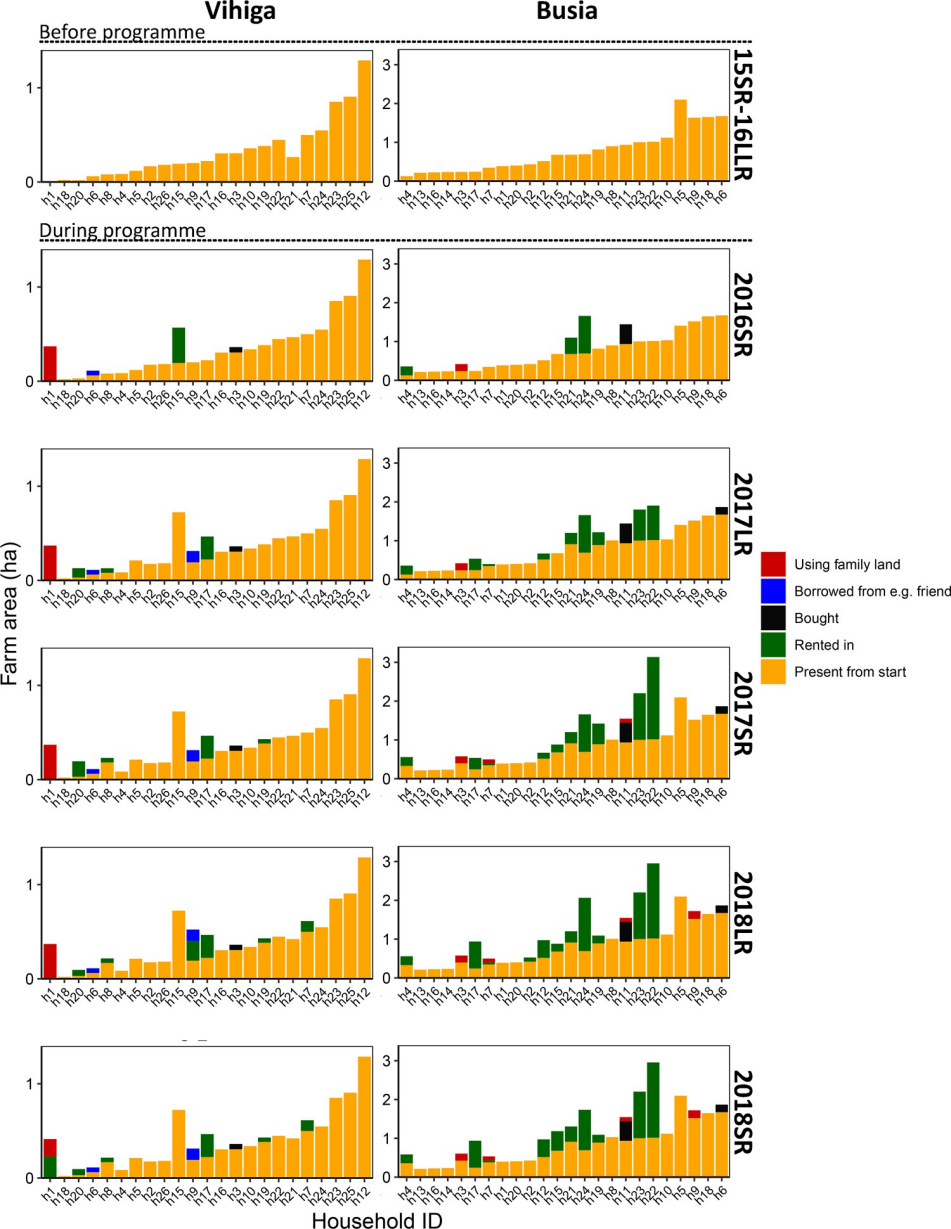
Farm area per household. Households were ordered according to their reported farm area in 2016LR, before the first season of the voucher programme. Some fields were reported in later seasons, while they were already owned in 2016LR.

For a number of households, value of produce remained low, in particular in Vihiga (households 3, 4, 6, 18 and 20, Fig 2), but also for some in Busia (households 7 and 16). These were mainly women-headed households with few household members and/or households with an ultra-small farm area of less than 0.2 ha (Fig 3). For them, the limited labour availability and the small farm area precluded a useful allocation of the inputs from the voucher, and as a result, part was given away or not used, as was reported in the monitoring survey. Household level production therefore only increased to a limited extent and sometimes was even less than the voucher value (S3 Appendix).

### Changes in farm area during the voucher intervention

Farm area increased for 8 out of 23 households in Vihiga and 14 out of 24 households in Busia after the voucher introduction (Fig 3). Most of this land was rented or family land that had been fallow before, the latter mainly in Busia. Only three households bought additional land. The initially small farms more often expanded their area than the larger farms, in particular in Vihiga (Fig 3 and S4 Appendix). Absolute increases in farm area were largest in Busia (Fig 3). Farmers reported that they wanted to make good use of the inputs and needed more land. For instance, Household 1 in Vihiga was a single-headed male household who initially owned only a small plot around his house. The farmer worked off-farm in a nearby town before the intervention and sold self-made charcoal. The voucher enabled him to borrow land from a relative who was living away in the city and to rent in land in later seasons. Thanks to this increase in farm area, he moved from being among the households with the lowest value of produce to the group of farmers with a high value of produce (Fig 2). In Busia, farmers increased their farm area mainly to boost production and sell the surplus. However, rent agreements were often informal and only held for single seasons, leaving farmers to search for new rental land. Landowners often refused to rent out their land for a subsequent season as they also wanted to profit from the high yields obtained with the voucher inputs. Only one household reduced the farm area as a field was given away to their son for building his house.

### Value of produce in relation to farm area

**Fig 4:**
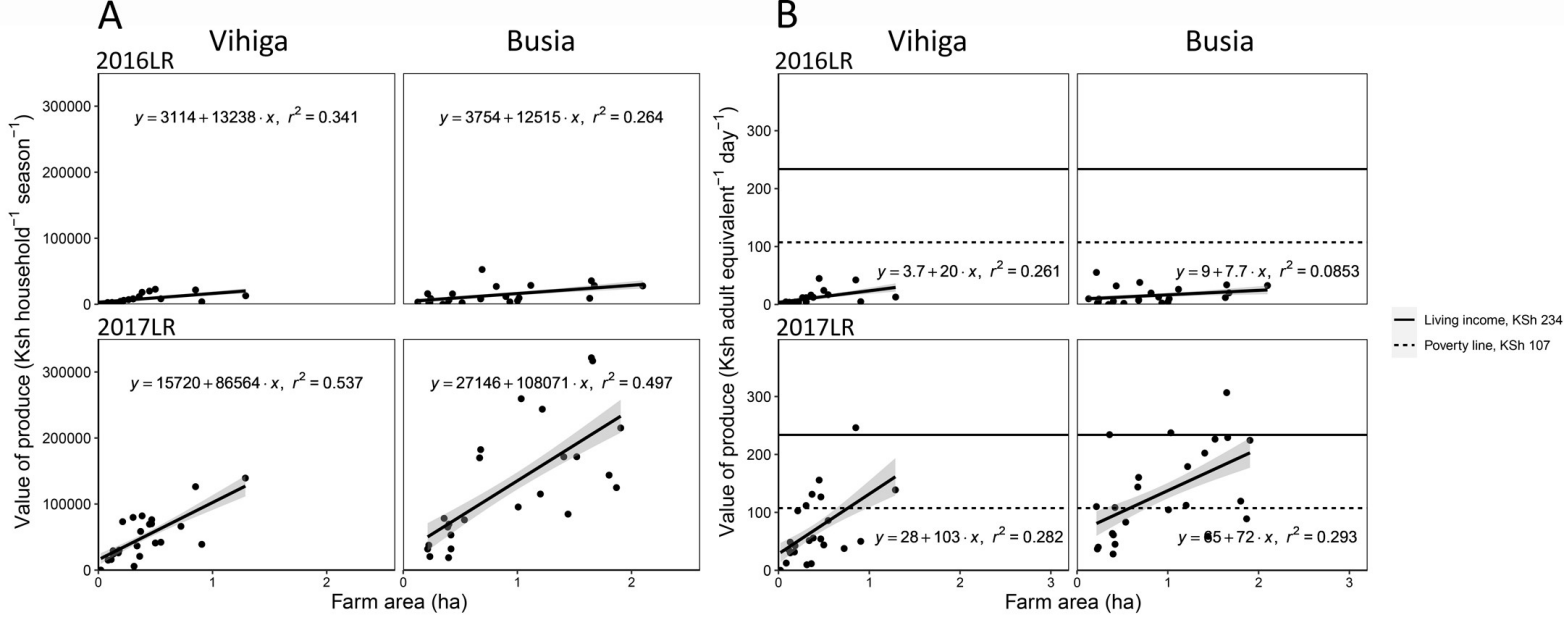
Total value of produce in Kenyan Shilling (KSh) per household per season (A) and the value of produce per adult equivalent per day (B) in relation to farm area during the long rains (LR) cropping season before the programme (2016) and during the programme (2017).

Total value of produce was related to farm area, and the relation was stronger after the start of the voucher intervention (2017LR in Fig 4A) than before the voucher was introduced (e.g. 2016LR in Fig 4A). Moreover, with the voucher, the difference in value of produce between households with a small and a large farm area increased (S5 Appendix). A similar pattern was observed when the value of produce was expressed per adult equivalent per day (Fig 4B), albeit with more variation, resulting from the variation in household size. The number of adult equivalents per household was higher in Busia (at a median of 4.6) than in Vihiga (at a median of 3.2), which reduced the differences between the two locations when expressed per adult equivalent. Some households obtained an above average-value of produce per unit area of land. These households did not necessarily obtain greater yields but planted almost their whole farm with the four crops that were part of the intervention.

## Discussion

Alleviating resource constraints through providing farmers with an input voucher resulted in an increase of maize yields at farm-level from 16% to 40–50% of the water-limited yield (Fig 1). Yet this large and immediate yield increase lifted only few households out of poverty (Fig 2). Even fewer households obtained a value of produce sufficient to reach a living income in Vihiga. The situation was better in Busia due to the farms being larger. With the introduction of the vouchers, land became a more strongly limiting factor for increasing the value of produce (Fig 4). The vouchers stimulated not only the intensification of agriculture by using more inputs, but also extensification, as farmers increased their farm area (Fig 3). However, with a much denser population, Vihiga harboured less options for expansion compared with Busia.

Our results showed that relieving cash constraints for purchase of inputs is a relatively easy measure to strongly increase input use and crop yields. We demonstrated that currently available technologies in western Kenya (e.g. varieties, fertilizer practices) are sufficient to reach 50% of the water-limited yield (Fig 1), which was proposed as a goal for reaching food self-sufficiency by 2050 in SSA [2]. This was achieved at the farm-level across farms in an area known for its high diversity within and between farms in terms of soil fertility and yield response [20, 39]. Reaching such a yield target would require substantial institutional changes in addition to the input voucher scheme tested in this study. Indeed, increased production through input subsidies brings a risk of overproduction and deflating prices, as was seen at national level with the Sasakawa Global 2000 programme in Ethiopia [11, 40]. Therefore, it is more effective to provide incentives to increase production as part of a package of policies [41], which also include e.g. improved infrastructure for increased market access, price protection, strategic grain reserves and dynamic subsidies that reduce if overproduction is looming or when markets become more functional [42, 43]. A production target should also consider other trade-offs, such as risks to the environment [44].

Providing a US$ 100 voucher each season may be expensive for African governments and the mixed results of recent input subsidy schemes in SSA should be considered [26] as these can shed a light on the effectiveness of such a scheme at scale. The aim of our study was not to assess returns on investment of the voucher. Our findings in Vihiga however show that for the 25% smallest farms, the total value of produce did not outweigh the value of the US$ 100 input voucher. This while for the remaining 75% of the farms in Vihiga and all farms in Busia, the value of produce outweighed the voucher (S3 Appendix). Agricultural production in the United States and Europe has been subsidized for decades with amounts that go beyond the US$ 100 voucher per cropping season. As an example, the EU direct income subsidy was about US$ 474 ha^-1^ year^-1^, including a re-greening subsidy, in The Netherlands in 2020 [45]. Farmers received on average a subsidy of US$ 270 ha^-1^ in Vihiga and US$ 118 ha^-1^ in Busia on a per hectare and per season basis. The difference in this comparison is due to the difference in farm area in both locations. Although this may not be a fair comparison with The Netherlands having a GDP that is 15 times larger than Kenya [46], it indicates the importance that other countries give to keeping agriculture profitable for farmers. In SSA, where agriculture contributes a large part of the economy, e.g. 34% for Kenya in comparison with less than 2% for the Netherlands [47], agricultural subsidy schemes may be important as agriculture is such a large part of the economy. Considering the difficult but needed transformation of smallholder agriculture in terms of increasing yields and farmer incomes [5], input subsidies could be part of a wider set of institutional changes that ensure that such a transformation is profitable for smallholder farmers. An option for SSA governments could be to start with initially smaller incentives such as temporary voucher schemes that, depending on farmers’ needs and government objectives, could support adoption of new options for a number of seasons (e.g. new varieties), and/or credit schemes similar to the ones provided by One Acre Fund. However, a fixed voucher scheme may have different outcomes than our flexible voucher where farmers could choose from a range of inputs to fulfil their diverse needs. Further research would be required to test such (temporary) voucher schemes, for instance by rolling out across more localities with a larger sample size and/or by comparing the approach against other possible interventions such as improved extension services or agricultural research and development.

Farm area limited the value of produce of farmers using the input voucher (Fig 4). Production without the voucher was less constrained by farm area, which may imply that intensifying production is currently not profitable and/or not within the reach for smallholder farmers given their current cash constraints, even if they have a relatively large farm area. Comparison between the poverty line and living income benchmarks illustrated the limited potential of cultivating basic staple crops on small plots in terms of achieving a decent living (Fig 2). This lack of prospects partly explains why smallholder farmers in current systems invest little in inputs and other technologies for increasing farm production and why farmers migrate to cities or other areas, for off-farm opportunities [7, 48, 49]. Our empirical results on the limitations of small farm areas are in line with Harris and Orr [6], who calculated household-level benefits of technologies tested on farm. Similarly, both Ritzema et al. [7] and Gassner et al. [50] showed in their scenario analysis that options for sustainable intensification would mainly benefit households with larger farm areas, while households with small farm areas remain food insecure and have limited financial benefits from such options. Income from farming can be increased by increasing farm areas through land reforms of existing farm land, which would require additional employment opportunities for those moving out of farming [5]. Without additional employment opportunities, the ultra-small farms would possibly be better off with a social safety net which does not, or only partly, focus on farming [51] than with an input voucher.

Farm area increased for more than one-third of participating farmers with the provision of a voucher, even in a densely populated area like Vihiga. Although increasing farm area for some households led to relatively large increases in value of produce, for none of the households this led to obtaining a living income. Facilitating secure land tenure arrangements could be an important role for national or local governments to foster the use of land that is currently not in use. This could enable for instance land-owners living elsewhere to rent out their land without the risk of losing their ownership rights, while those who are renting can increase production [52]. In other areas where land is relatively more abundant, such as in Busia, current fallow land can be used to increase farm area, as we found. The use of fallow land in Busia, partly fits in a wider trend in SSA of increasing cultivated land area, resulting in extensification instead of (sustainable) intensification on land currently in use [3, 53]. However, these trends of extensification are often the result of the increasing smallholder farming population and new groups of large land owners going into farming [54]. Cultivated area per farm however, generally decreases in current farming systems due to land fragmentation and population pressure [55, 56]. The increase in cultivated area per farm that we found, may therefore be a specific result of the input voucher.

Additional research is required to assess how a living income from farming could be attained through changes in farm area and/or adjusting the cropping system, e.g. cultivating more profitable crops or reaching higher yield levels [57]. High-value crops could be included in a voucher or subsidy scheme for increased household level income and diversified production. In this study we focussed on the main crops cultivated and those important for food security (e.g. maize, beans). For some households, legumes were an important part of their value of produce, more than maize. Crop diversity also allows crop rotations [58] and benefits household nutrition [59]. Other crops like vegetables can be more profitable, but often are much more perishable and management requires more attention than grain crops [60].

Our study was conducted in western Kenya, which is representative of the East African highlands in terms of the bimodal rainfall pattern and deep soils, resulting in a favourable agroecological potential compared to many other regions of SSA [61]. Most inputs were relatively easily available and to some extent, farmers were accustomed to applying mineral fertiliser and sowing improved varieties. This may be due to a long history of promoting these inputs by the Kenyan government, NGOs and, in recent years, by One Acre Fund. One Acre Fund was already active within our location and remained active throughout the programme with a similar intensity. This context favouring input use is quite different from many regions in SSA, including for instance neighbouring Uganda, where little mineral fertiliser is applied to food crops [62]. In such cases where farmers lack experience of fertiliser use [63], it may require more effort to increase farmers’ knowledge and encourage uptake through e.g. on-farm demonstrations and learning activities.

The detailed empirical work of this study, including crop yield sampling in all the fields of the 47 participating farmers, meant that we could not work with a larger sample of farmers. However, the purposeful selection of a diverse group of farmers, following Giller et al. [23], provides confidence that yields and production results are representative for the smallholder farming systems in western Kenya. Grain yields before the intervention were based on farmer-reported production per field while during the intervention they were based on measured crop cuts. The farmer-reported maize yields in 2015SR (median 1350 kg ha^-1^) were in line with reported yields by local counties in the period 2012–2014 at an average 1600 kg ha^-1^ for Vihiga and 1450 kg ha^-1^ for Busia [64]. Farmer-reported yields in 2016LR (850 kg ha^-1^) were much lower due to exceptionally poor rainfall, which is why we mainly considered 2015SR as a reference season for yields before the programme (i.e. the 16% of the water-limited yield). Farmer-reported maize yields before the programme (measured field sizes) were lower than the overall average yields reported by Sheahan et al. [65] for maize growing area across Kenya. This average however also included locations with better potential for maize production (e.g. Trans Nzoia and Nakuru) and was based on farmer-reported production and farmer-reported field sizes. Farmer-reported field sizes are notoriously prone to errors [66], which is why we measured field sizes. Farmer-reported rates of N input use in our study were comparable to those reported by Sheahan et al. [65]. Farm-level yields obtained during the programme (measured) aligned with yields from earlier field-level experimental work in western Kenya by Njoroge et al. [17] and Vanlauwe et al. [20] and were larger than the relatively poor yields described by Roobroeck et al. [67], in particular during the long rains cropping seasons. The immediate response to increased input use as found in our study was similar to the response found by Njoroge [68] in western Kenya, when they applied full fertilisation after eight seasons of no or unbalanced fertilisation. During monitoring of input use we did not observe any new subsidy schemes or other changes in input availability in our research locations. Apart from the input vouchers we provided, crop inputs were mainly obtained from local agro-input stores and One Acre Fund, which both were already present and active during the two seasons before the programme. Yield increases during the programme, as compared with before the programme, were therefore seen as realistic given the increased use of fertilisers and improved varieties as a result of the input voucher.

Our results paint a positive picture of household-level financial gains from agriculture. We used ‘value of produce’ as an estimate for income and input costs were not subtracted, as most was provided through the voucher. Including the voucher as a cost, would result in a negative income for the smallest farms (S3 Appendix). Costs for other inputs besides the voucher inputs were also not included, so that in reality, income from farming would be less. On the other hand we only included the four main crops, leaving out crops like trees, Napier grass and vegetables. Napier grass and vegetables were mainly cultivated by the larger farms, while trees were grown on land unsuitable for arable crops (e.g. rocky outcrops, waterlogged areas). Hence, overall, our results provide an important insight in the impact of a US$ 100 input voucher on household value of produce and limitations by farm area.

## Conclusions

Increasing food demand in SSA, as a result of the burgeoning population, will require substantial changes in farming systems to increase production of basic staple crops. Smallholder farmers, who currently supply most of the national food needs but achieve low yields and mostly live in poverty, will need smart incentives and other support if they are to be part of providing for these future food needs. In this study we tested such an incentive for increasing production: a US$ 100 input voucher per season, to increase yields and farmer income. Our results showed that providing a US$ 100 input voucher increased maize yield from 16% to 40–50% of the water-limited yield, which was insufficient to provide a living income for most farmers. Therefore, in current smallholder farming systems, crop yields are mainly limited by cash constraints at household level and not by technological constraints. Farm area was an important limiting factor for value of produce when input use increased with the introduction of the voucher. For future farming systems therefore, an increase in the farm area per farm is essential to provide smallholder farmers a viable pathway out of poverty. As a consequence, changes will be required, such as creating off-farm employment opportunities, social safety nets and land reforms to create opportunities for many current farmers that are now ‘hanging in’ on unviable small farms.

## Supporting information

S1 Appendix Detailed farm characterization survey to assess the differ fields in the farm, farm size, input use, yields and production the two seasons before the programme. (DOCX)

S2 Appendix Grain prices in 2018 for maize (A) and bush bean, groundnut and soybean (B) based on weekly price observations in Busia (Matayos market) and Vihiga (Luanda market)
(DOCX)

S3 Appendix Total value of produce (Ksh) per household per season. Households are ordered according to their value of produce per adult equivalent per day as shown in Fig 2. Differences in order are therefore caused by the household size per household, that was used to calculated the adult equivalents per household.
(DOCX)

S4 Appendix Additional farm area and farm area present from the start as percentage of the total farm area per household per season. Households were ordered according to their initial farm area in 2016SR, see also Fig 3.
(DOCX)

S5 Appendix Value of produce per household per season in relation to farm area. Seasons 2015SR and 2016LR were before the programme while the following seasons were during the programme.
(DOCX)
