# Supplementary material for: Narrowing yield gaps does not guarantee a living income from smallholder farming–an empirical study from western Kenya
Source: PLoS One. 2023 Apr 20;18(4):e0283499. doi: 10.1371/journal.pone.0283499 (PMC10118150; doi:10.1371/journal.pone.0283499)
Supplement: S1 Appendix — (DOCX) [file pone.0283499.s001.docx]

S1 Appendix

Detailed farm characterization survey to assess the differ fields in the farm, farm size, input use, yields and production the two season before the programme.

**Part A: General information**

^1^If no household ID is known yet (new household), give new ID, starting with V-100 for Vihiga and B-100 for Busia and counting upwards from there (next household after B-100, is B-101).

|  | A.1. General information | |  |  |
| --- | --- | --- | --- | --- |
| *Household –ID^1^* |  | |  |  |
| *Date (dd/mm/yyyy)* |  | |  |  |
| *County* |  | |  |  |
| *Region* |  | |  |  |
| *Ward* |  | |  |  |
| *Village* |  | |  |  |
| *Name Interviewer* |  | |  |  |
|  | Latitude decimal degrees | | Longitude degrees | |
| *Homestead!! GPS code* |  | |  | |
| *Name of the respondent* |  |  |  |  |
| *Gender* |  |  |  |  |
| *Age* |  |  |  |  |
| *Position in household^2^* |  |  |  |  |
| *Household type^3^* |  |  |  |  |
| *Mobile phone number* |  |  |  |  |

| **^2^Position in household:**  1= Household head  2= Joint household head  3= Spouse of head  4= Other family member  5= Other, none family member | **^3^Household type**  1= Live together  2= Single, divorced or widowed  3= Spouse works away  4= Other adult in charge  5= Child headed |
| --- | --- |

**A.2. Member of a group**

Are you member of a farmers group/cooperation/SACCO/etc? Name:_________________________

If yes; Which benefits do you receive? ___________________________________________

Did you receive any inputs (e.g. fertilizer, seeds) through one of these groups in 2015B or 2016A? Yes/No, If yes, what amounts did you receive for those seasons and what did you have to pay?____

_________________________________________________________________________________

**Part B: Household Roster** *Include only members who live there at least 3 months per year.*

**B.1. How many people are there in your household ____________**

| ID |  | | Number of male | | Number of female | Highest Level of Education (code a) | How many are working on the farm? If not full time, note percentage of time. |
| --- | --- | --- | --- | --- | --- | --- | --- |
| 1 | Respondent | |  | |  |  |  |
| 2 | Household head | |  | |  |  |  |
|  | *People per age group* | |  | |  |  |  |
| 3 | 0 – 16 | |  | |  |  |  |
| 4 | 17 – 35 | |  | |  |  |  |
| 5 | 36 – 60 | |  | |  |  |  |
| 6 | Over 60 | |  | |  |  |  |
|  | |  | | **a) HIGHEST LEVEL OF EDUCATION** | | | |
|  | |  | | 1= Can not read or write  2= Can read and write  3= Primary  4= Secondary  5= Post-secondary | | | |

**Selection of fields and soil sampling**

Definition of field:

A field in this study is an area of land with the same cropping systems and same soil management (tillage, fertilizer, manure). For instance if there are three terraces (same size in this example) adjacent to each other, all cultivated with maize and where the upper terrace always receives 10 wheelbarrows of manure and the lower two terraces both receive four wheel barrows. Then the upper terrace is considered as a separate field and the two lower terraces are seen as one field together.

Sketch with location of sampled field (on empty next page)

- For every farm make a drawing indicating the position of each field as compared to the homestead and other landmarks (roads, trees, rocks, etc.). Include all fields owned or rented-in by the household (including the homestead grazing field, home garden, tea or sugar fields, tree fields, etc).
- Take the GPS coordinates (longitude/latitude in decimal degrees) of each field and record on sketch as well as sample register

Crops of interest

Soil samples should be taken from all fields cultivated by field crops or that could easily be cultivated. E.g. maize, beans, Napier grass, sweet potato, soybean, groundnut, sorghum and banana should be sampled. Field with trees, tea, sugar, very small fields, and fields that have mainly other crops like coffee don’t need to be sampled. Home gardens need to be sampled. A fallow or grazing field that is sometimes cultivated should be sampled. If a farmer has more than 6 fields for sampling, still take a sample of all fields, but make a note of which fields could be excluded for analysis (less important fields e.g. fallow fields, smaller fields or fields that are regarded as not important by the household)

Soil sampling

- Take soil samples from the top 20 cm of soil at 6 different places in a field
- Collect all samples in a basin and mix thoroughly to make a composite sample
- Per field fill two ziplock bags (5-6 inch wide) with soil. Make paper labels with Farm ID and Field number to put inside the bag.Bring samples to station in Maseno for drying and shipment to the lab !!! Sort the samples from one farm into one bag !!!

Field sketch: Household-ID_______________________

| FARM ID | FIELD NUMBER | GPS COORDINATE (longitude, latitude; decimal degrees) | Elevation |
| --- | --- | --- | --- |
|  |  |  |  |
|  |  |  |  |
|  |  |  |  |
|  |  |  |  |
|  |  |  |  |
|  |  |  |  |
|  |  |  |  |
|  |  |  |  |
|  |  |  |  |
|  |  |  |  |
|  |  |  |  |
|  |  |  |  |
|  |  |  |  |
|  |  |  |  |
|  |  |  |  |
|  |  |  |  |

**Part G: Field characterization** (see soil sampling protocol for details on soil sampling)

| Field ID  (number) | *Soil sample taken?^2^ (y/n)* | *Texture analysis needed? (y/n)*^2^ | *Location*  *(GPS-coordinates*)^2^ | *Soil type (e.g. sandy, loamy, clay, dark, red)*^2^ | Field size and unit  (measured^3^) | Slope class  F=flat  S=steep  V=very steep | Visible erosion  1=none  2=moderate  3=severe | Geographical location (hillside, valley, etc.) | Erosion measures (stone bunds, ditches, mulching, etc.) |
| --- | --- | --- | --- | --- | --- | --- | --- | --- | --- |
|  |  |  |  |  |  |  |  |  |  |
|  |  |  |  |  |  |  |  |  |  |
|  |  |  |  |  |  |  |  |  |  |
|  |  |  |  |  |  |  |  |  |  |
|  |  |  |  |  |  |  |  |  |  |
|  |  |  |  |  |  |  |  |  |  |
|  |  |  |  |  |  |  |  |  |  |
|  |  |  |  |  |  |  |  |  |  |
|  |  |  |  |  |  |  |  |  |  |

^2^ *Soil sample taken, Texture analysis needed,* and *Soil type* only need to be noted for fields of which a soil sample is taken be filled in after the field visit, after the samples have been dried. *Location* can be taken from the soil sample sheet, except for those fields that are not sampled yet.

^3^ The area of larger plots (more than 20m x 20m) need to be measured using a hand-held GPS Smaller fields must be measured manually with a tape measurer.

^4^ Shaded data fields are researchers observations and should not be asked to the farmer but measured or observed by the researcher while in the field.

| Field ID | Field fertility farmer estimate 1=poor, 2=medium, 3=good | Last time fallow?  (season) | Distance from homestead (min. farmer est.)? | Land ownership  o=owned, r=rented, c=communal | Crop rotation  Crops:  2014B | 2015A | Type of land preparation  (2015B and 2016A) | Other comments/ observations for this field? |
| --- | --- | --- | --- | --- | --- | --- | --- | --- |
|  |  |  |  |  |  |  |  |  |
|  |  |  |  |  |  |  |  |  |
|  |  |  |  |  |  |  |  |  |
|  |  |  |  |  |  |  |  |  |
|  |  |  |  |  |  |  |  |  |
|  |  |  |  |  |  |  |  |  |
|  |  |  |  |  |  |  |  |  |
|  |  |  |  |  |  |  |  |  |
|  |  |  |  |  |  |  |  |  |

**2015B: Input use and yields short rains** (fill only for fields cultivated with crops during this season, include fields with Napier grass and other perennials)

| Field ID | **Crop**(s) grown  (if intercropped, name all crops and indicate relative shares, e.g. 80% maize / 20% beans) | **Variety** per crop  (if variety name is not known, ask for type, e.g. hybrid, local, improved) | Mineral **fertiliser**(s) applied?  (If yes specify:  type, amount+unit, price. If no, leave blank) | **Manure** applied  Amount+unit (bags, buckets, etc.) | **Other inputs** used?  Biocides, inoculants, etc. (type, amount, price) | **Residue use**?  % left in the field?  % collected and fed?  % incorporated  % sold? % burned?  Specify per crop | Amount **harvested** per crop? (give unit, e.g. in kg, gorogoro, 100 kg bags)  Amount **sold** + price? Where was it sold? |
| --- | --- | --- | --- | --- | --- | --- | --- |
|  |  |  |  |  |  |  |  |
|  |  |  |  |  |  |  |  |
|  |  |  |  |  |  |  |  |
|  |  |  |  |  |  |  |  |
|  |  |  |  |  |  |  |  |
|  |  |  |  |  |  |  |  |
|  |  |  |  |  |  |  |  |
|  |  |  |  |  |  |  |  |
|  |  |  |  |  |  |  |  |

**2016A: Input use and yields long rains** (fill only for fields cultivated with crops during this season, include fields with Napier grass and other perennials)

| Field ID | **Crop**(s) grown  (if intercropped, name all crops and indicate relative shares, e.g. 80% maize / 20% beans) | **Variety** per crop  (if variety name is not known, ask for type, e.g. hybrid, local, improved) | Mineral **fertiliser**(s) applied?  (If yes specify:  type, amount+unit, price. If no, leave blank) | **Manure** applied  Amount+unit (bags, buckets, etc.) | **Other inputs** used?  Biocides, inoculants, etc. (type, amount, price) | **Residue use**?  % left in the field?  % collected and fed?  % incorporated  % sold? % burned?  Specify per crop | Amount **harvested** per crop? (give unit, e.g. in kg, gorogoro, 100 kg bags)  Amount **sold** + price? Where was it sold? |
| --- | --- | --- | --- | --- | --- | --- | --- |
|  |  |  |  |  |  |  |  |
|  |  |  |  |  |  |  |  |
|  |  |  |  |  |  |  |  |
|  |  |  |  |  |  |  |  |
|  |  |  |  |  |  |  |  |
|  |  |  |  |  |  |  |  |
|  |  |  |  |  |  |  |  |
|  |  |  |  |  |  |  |  |
|  |  |  |  |  |  |  |  |
