# Supplementary material for: Narrowing yield gaps does not guarantee a living income from smallholder farming–an empirical study from western Kenya
Source: PLoS One. 2023 Apr 20;18(4):e0283499. doi: 10.1371/journal.pone.0283499 (PMC10118150; doi:10.1371/journal.pone.0283499)
Supplement: S2 Appendix — Grain prices in 2018 for maize (A) and bush bean, groundnut and soybean (B) based on weekly price observations in Busia (Matayos market) and Vihiga (Luanda market) (DOCX) [file pone.0283499.s002.docx]

S2 Appendix

Grain prices in 2018 for maize (A) and bush bean, groundnut and soybean (B) based on weekly price observations in Busia (Matayos market) and Vihiga (Luanda market).


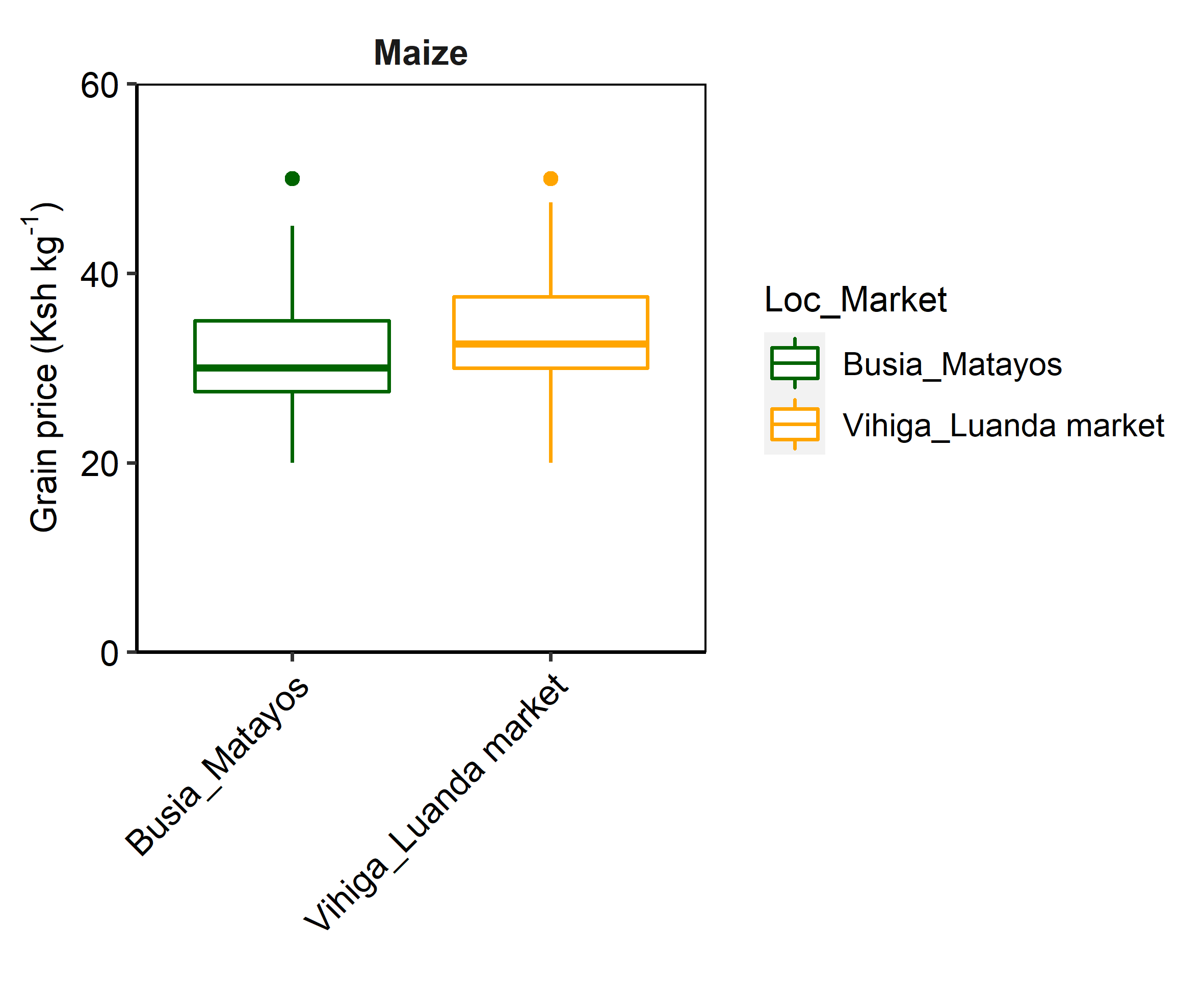

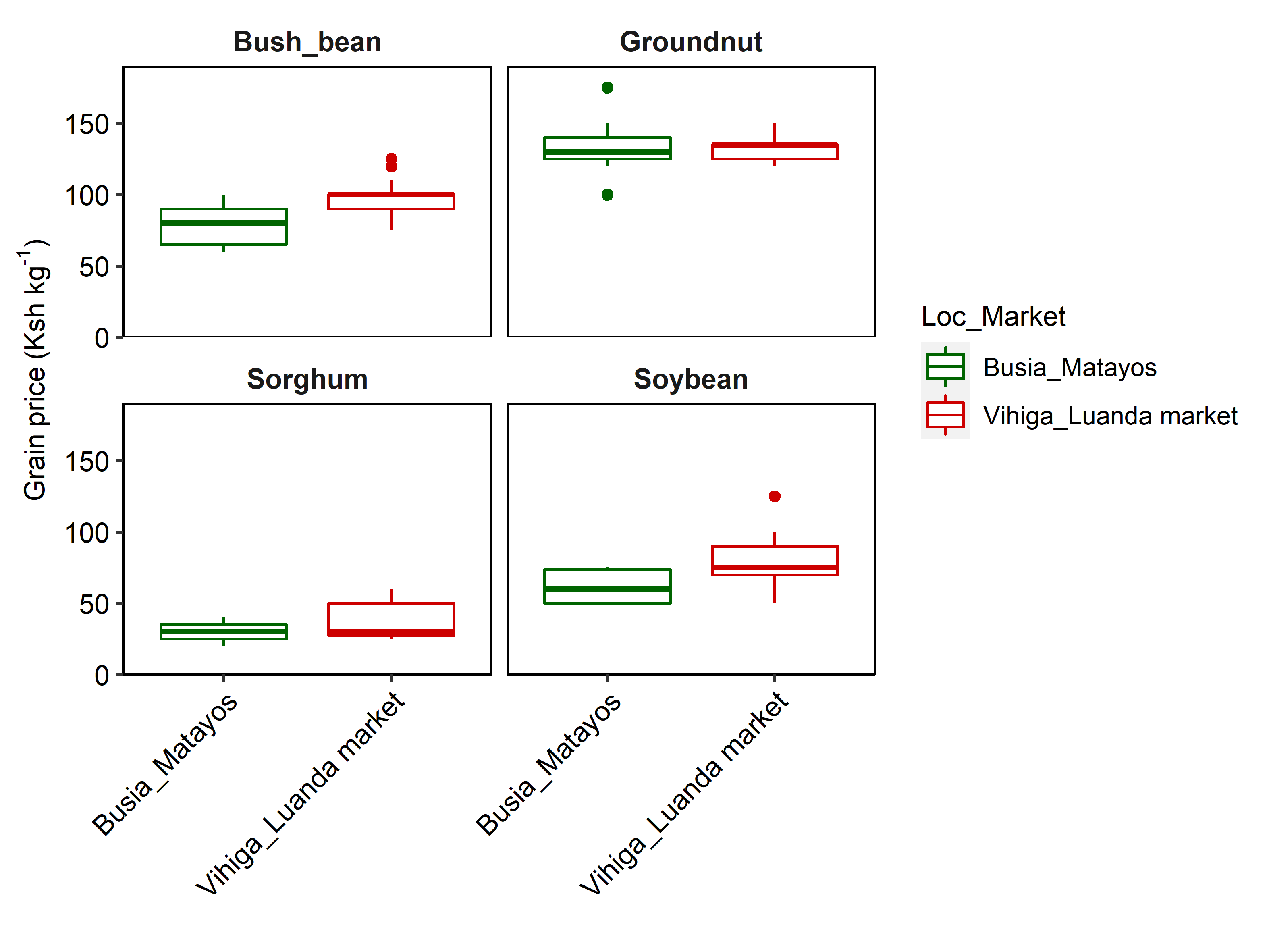


**A**

**B**
