# Supplementary material for: Narrowing yield gaps does not guarantee a living income from smallholder farming–an empirical study from western Kenya
Source: PLoS One. 2023 Apr 20;18(4):e0283499. doi: 10.1371/journal.pone.0283499 (PMC10118150; doi:10.1371/journal.pone.0283499)
Supplement: S3 Appendix — Households are ordered according to their value of produce per adult equivalent per day as shown in Fig 2. Differences in order are therefore caused by the household size per household, that was used to calculated the adult equivalents per household. (DOCX) [file pone.0283499.s003.docx]

S3 Appendix


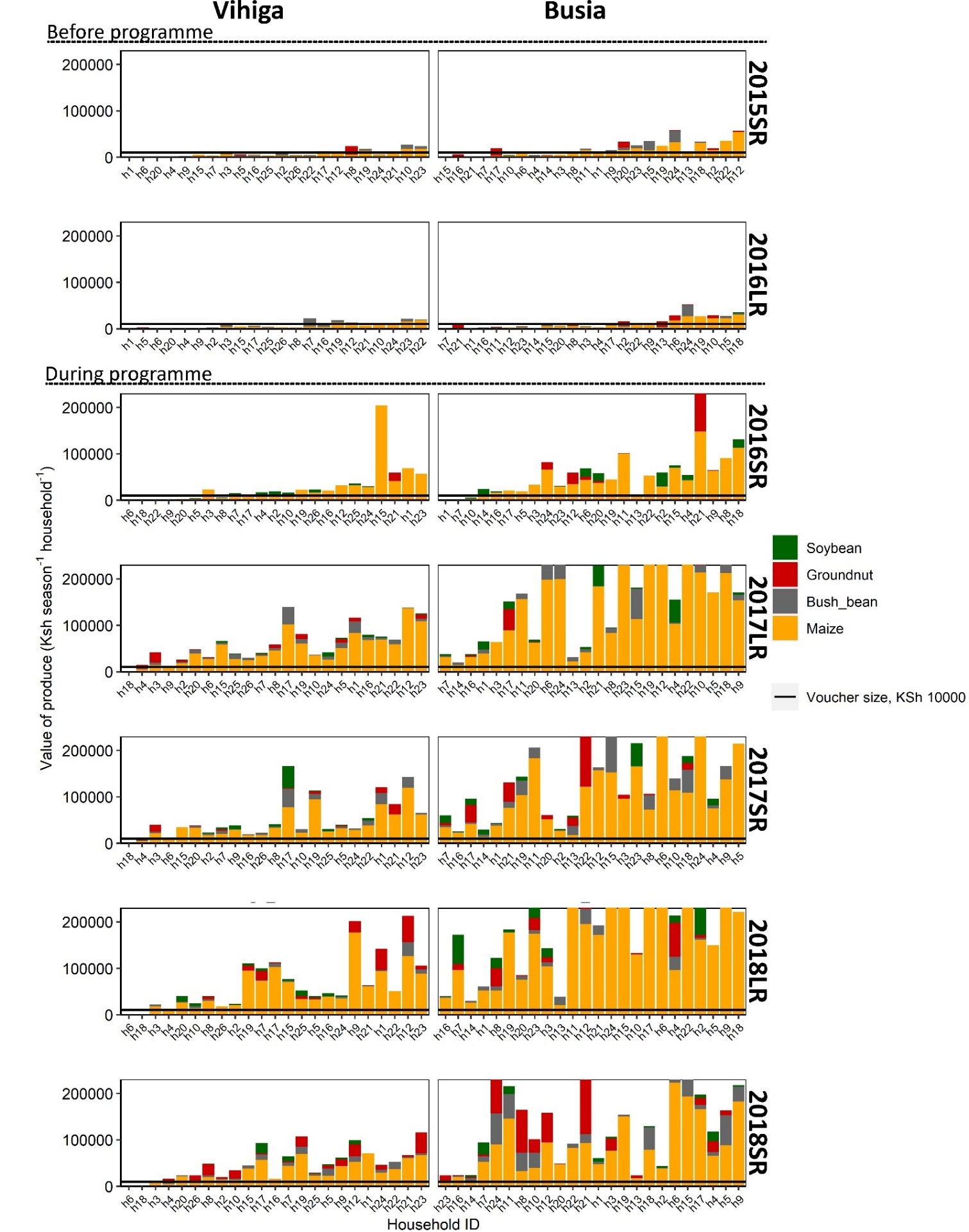


Total value of produce (Ksh) per household per season. Households are ordered according to their value of produce per adult equivalent per day as shown in Fig. 2. Differences in order are therefore caused by the household size per household, that was used to calculated the adult equivalents per household.
