# Supplementary material for: Narrowing yield gaps does not guarantee a living income from smallholder farming–an empirical study from western Kenya
Source: PLoS One. 2023 Apr 20;18(4):e0283499. doi: 10.1371/journal.pone.0283499 (PMC10118150; doi:10.1371/journal.pone.0283499)
Supplement: S4 Appendix — Households were ordered according to their initial farm area in 2016SR, see also Fig 3. (DOCX) [file pone.0283499.s004.docx]

S4 Appendix


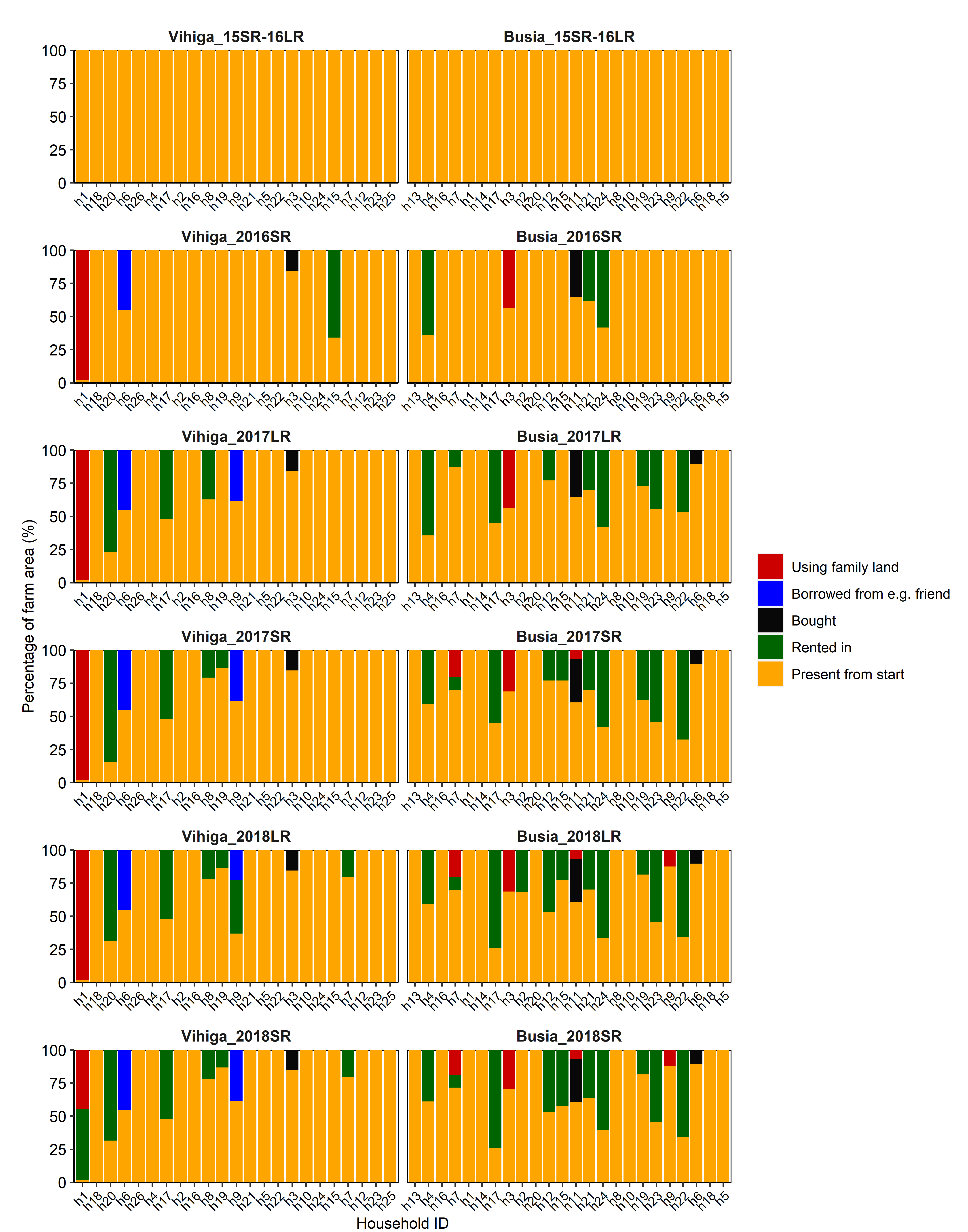


Additional farm area and farm area present from the start as percentage of the total farm area per household per season. Households were ordered according to their initial farm area in 2016SR, see also Fig. 3.
