# Supplementary material for: Narrowing yield gaps does not guarantee a living income from smallholder farming–an empirical study from western Kenya
Source: PLoS One. 2023 Apr 20;18(4):e0283499. doi: 10.1371/journal.pone.0283499 (PMC10118150; doi:10.1371/journal.pone.0283499)
Supplement: S5 Appendix — Seasons 2015SR and 2016LR were before the programme while the following seasons were during the programme. (DOCX) [file pone.0283499.s005.docx]

S5 Appendix


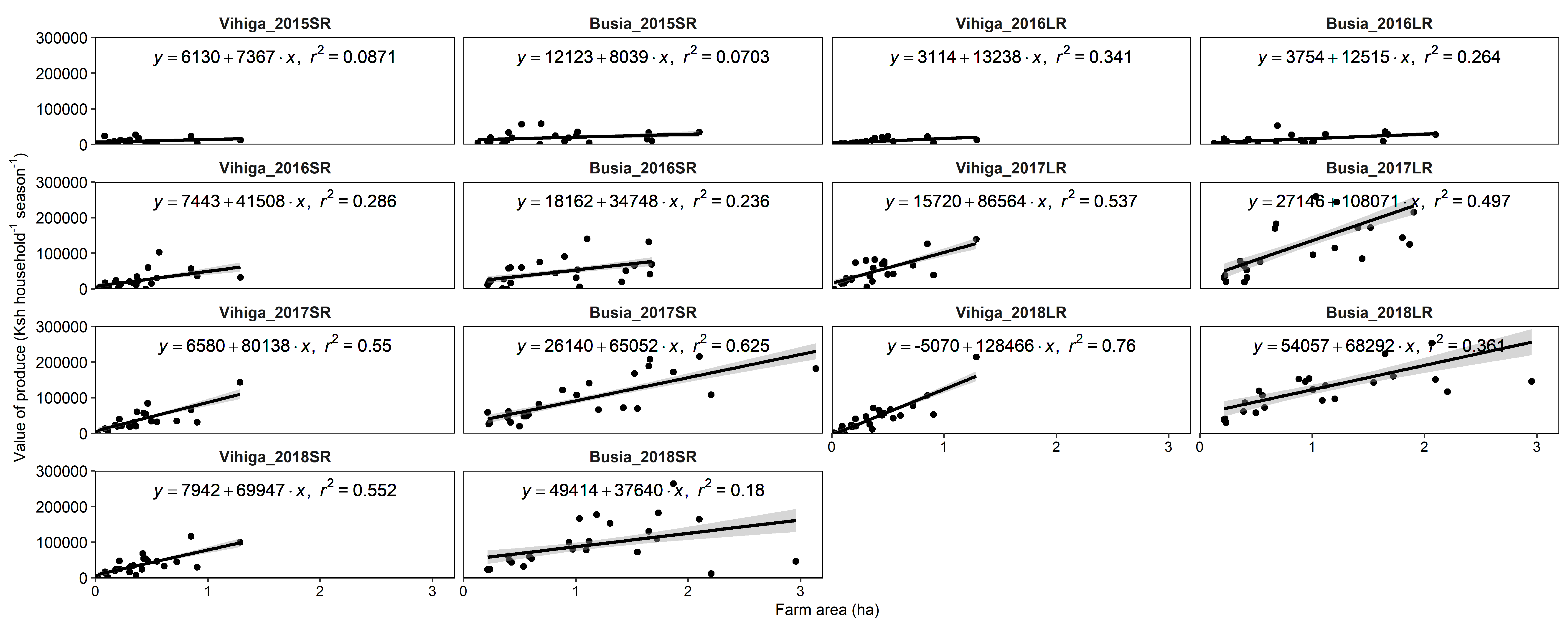


Value of produce per household per season in relation to farm area. Seasons 2015SR and 2016LR were before the programme while the following seasons were during the programme.
